# CHRONIC AUTOIMMUNE URTICARIA: WHERE WE STAND?

**DOI:** 10.4103/0019-5154.55640

**Published:** 2009

**Authors:** C L Goh, K T Tan

**Affiliations:** *From the Department of Dermatology, National Skin Center, Singapore.*

**Keywords:** *Chronic autoimmune urticaria*, *urticaria*, *chronic urticaria*

## Abstract

It is well-recognized that 30-40% of chronic idiopathic urticaria is autoimmune in nature. Chronic autoimmune urticaria is caused by anti-FcεRI and less frequently, by anti-IgE autoantibodies that lead to mast cell and basophil activation, thereby giving rise to the release of histamine and other proinflammatory mediators. Activation of the classical complement pathway and formation of C5a are important in dermal mast cell activation. C5a is also a neutrophil and eosinophil chemoattractant. Chronic autoimmune urticaria has been found to be associated with autoimmune thyroid disease. The autologous serum skin test is used as a screening test for chronic autoimmune urticaria and has a sensitivity and specificity of about 70 and 80%, respectively. The current gold standard diagnostic test is the basophil histamine release assay. The treatment of chronic autoimmune urticaria, as in chronic idiopathic urticaria, is with H1 antihistamines. Oral corticosteroids may be used during acute flares. Refractory cases have been shown to respond to cyclosporine and other immunomodulators. The prevalence of chronic autoimmune urticaria in Singapore is similar to that reported in Western countries at about 42%. The presence of thyroid autoimmunity appears to be higher than reported, with 22.5% of patients with chronic idiopathic urticaria here, exhibiting presence of thyroid autoantibodies.

## Introduction

This is a review article of an update on chronic autoimmune urticaria.

Chronic urticaria (CU) is defined as urticaria persisting daily or almost daily for more than six weeks. It causes severe impairment on the quality of life.[1] CU includes physical urticaria, chronic “idiopathic” urticaria (CIU), and urticarial vasculitis. It is important to recognize patients with physical urticaria, as the need for investigation and treatment modalities differ from patients with CIU or urticarial vasculitis.

Although in many patients with CU the disease remains “idiopathic”, in recent years, a significant number of patients with non-physical CU have been reported to have an autoimmune basis for their urticaria. This has important implications when investigating CIU and when planning treatment for patients with CIU.

Hence, if we eliminate physical urticaria and urticarial vasculitis from all patients with CU, the remainder can be divided into chronic autoimmune urticaria (CAU) (45%) and CIU (55%). The autoimmune subgroup is associated with the IgG anti-IgE receptor alpha subunit in 35-40% of the patients, and IgG anti-IgE in an additional 5-10%. These autoantibodies have been shown to activate blood basophils and cutaneous mast cells *in vitro,* with augmentation of basophil activation by complement and release of C5a. Unfortunately antibody binding tests (immunoblot and ELISA) can yield positive tests in many autoimmune diseases, normal subjects, or patients with other forms of urticaria, but most such sera are nonfunctional and cannot be used to diagnose CAU. At this point in time, the gold standard for detecting clinically relevant autoantibodies to FcεRI is the functional *in vitro* donor basophil histamine release assay. Activation of basophils or mast cells causing histamine release is quite specific to chronic urticaria and defines the autoimmune subgroup. Although pathogenicity is not formally proven, the antibodies cause whealing upon intradermal injection, and removal of the autoantibody leads to remission.[2] CAU has been reported to occur in children as well.[3]

## What is Chronic Autoimmune Urticaria?

It is now recognized that approximately 30-40% of patients with CIU have histamine-releasing autoantibodies directed against either the high-affinity IgE receptor, or less frequently, IgE. The presence of autoantibodies may be important clinically in a small group of severely affected, treatment-resistant patients, where immunomodulatory treatments may be helpful.

## History of CAU

Although the concept of CAU as an autoimmune disease is fairly new, Rorsman's report in 1962[4] that in CU, “antigen-antibody reactions bring about degranulation of leucocytes” was followed by the observation that circulating thyroid autoantibodies, and more controversially, dysfunctional thyroid disease, showed a significant association with CIU. Leznoff later proposed a “syndrome” of autoimmune thyroid disease and CU.[5]

Hide *et al.,*[6] in 1993 and Nimii *et al.,*[7] in 1996 showed that autoantibodies against the high affinity IgE receptor or less commonly IgE itself were the cause of whealing in some patients with CU. The findings were subsequently independently confirmed by several groups.[8,9]

## Pathogenesis of CAU

Although a large number of naturally occurring immunological and nonimmunological agents are known to be capable of activating dermal mast cells, only allergen-specific IgE reactions, anti-IgE and anti-FcεRI autoantibodies, and complement C5a are of significant importance in CU.

Dermal mast cells secrete preformed mediators including histamine, proteases, interleukin-1, and tumor necrosis factor-α (TNF-α). These cytokines cause increased expression of adhesion molecules by the endothelium of the post capillary venules. *De novo* synthesized mediators include leukotrienes, prostaglandins, cytokines, and chemokines resulting in leukocyte recruitment including eosinophils, which characterize the late phase reaction.[10] An MHC class II-dependent signaling pathway enables mast cells to behave as antigen presenting cells,[11] which by activating T cells are able to maintain the duration of the wheals.

Little or no anti-FcεRI immunoreactivity is found in the IgM fraction. Isolation of IgG high affinity IgE receptor autoantibody subclasses using protein G affinity chromatography in patients' sera has shown that a majority of the histamine-releasing activity co-localizes with the IgG subclasses IgG1 and IgG3[12] and similar results are also reported on using immunoblotting.[13] Occasionally sera contained histamine-releasing IgG4, but no histamine-releasing autoantibody activity was found in subclass IgG2.

The high affinity IgE receptor FcεRI consists of four peptide chains: intracellular γ1, γ2, and β chains and an α-chain with an extracellular portion, which bears the binding site for IgE. Competitive inhibition studies, using a human recombinant α-chain of FcεRI, have established that the binding sites for IgG anti-FcεRI autoantibodies are located on the external portion of the α-chain.[14] Binding can occur on at least two domains in this region of the α-chain. These autoantibodies cross-link and dimerise FcεRI, leading to mast cell basophil activation and release of histamine and other mediators.

Up to 5-10% of patients with CAU have autoantibodies with specificity for IgE itself. These combine with and cross-link receptor-bound IgE, and by dimerising mast cell-bound IgE, evoke the release of histamine. Removal of IgE from mast cells or basophils by lactic acid stripping renders these autoantibodies inactive.

Anti-FcεRI and anti-IgE autoantibodies are functional, leading to mast cell and basophil activation and release of histamine and other proinflammatory mediators. The molecular cascade of events linking the autoantibody-antigen reaction with mediator synthesis and release has not been fully characterized.

### Role of complement

Complement depletion or inactivation diminishes histamine release evoked by anti-FcεRI autoantibodies *in vitro.*[15] Subsequent studies established the involvement of C5a and the classical complement activation pathway.[16] This finding explains the otherwise puzzling observation that symptoms and signs consequent upon interaction between anti-FcεRI autoantibodies, and their antigenic targets on mast cell membranes are limited to the skin, as pulmonary mast cells are devoid of C5a receptors. Activation of the classical complement pathway and formation of C5a is important in bringing about dermal mast cell activation. C5a is also a neutrophil and eosinophil chemoattractant, giving rise to the observed accumulation of these cells in the lesional skin.

## Diagnostic Tests for CAU

It is often not possible to distinguish CAU from those without autoantibodies, clinically or histologically. The autologous serum skin test (ASST) is used as a screening test. Gratten *et al.,* reported that the histological features of a positive ASST resemble an IgE-mediated late phase reaction.[10]

The ASST is a useful tool for picking up patients with circulating wheal-producing factors in CIU. However, its specificity as a screening test for the presence of functional anti-FcεRI is low, and confirmation by demonstration of histamine-releasing activity in the patient's serum is needed for establishing this diagnosis. The ASST is at best 80% sensitive and specific. Improved screening tests are being sought.

### Autologous serum skin test

The ASST was first described by Grattan[18] and subsequently characterized by Sabroe *et al.*[19] Briefly, serum is obtained from the patient, preferably with active urticaria. This is injected intradermally into the same patient's uninvolved skin, and the injected skin is examined for wheal formation 30minutes later. Positive and negative controls with intradermal histamine and saline injections are carried out on the adjacent skin. The test is positive if the serum-induced wheal is at least 1.5mm greater than the saline wheal. The ASST has a sensitivity of about 70% and specificity of about 80%. However, experience is required to produce reliable results.[20] In a recent study, Asero *et al.,* found that out of 78 patients with CIU, 35% had a positive ASST, but only 25% was positive for histamine-releasing activity against donor basophils.[21] Presumably, the “false” positive ASSTs were due to the presence of other wheal-inducing factors in the serum, such as, the mast cell specific factor or vasoactive kinin-like products released during the course of coagulation. Thus, there are marked differences between *in vivo* ASST findings and the results of the histamine release tests *in vitro*, as only a proportion of the ASST positive sera are able to release histamine from donor basophils *in vitro*. The ASST should be considered a crude screening test and should not be used as a specific test for the detection of circulating autoantibodies.[22]

The ASST positivity has also been reported to correlate with the duration of CIU attacks and with disease severity,[23] although it was also reported that the test was persistent in some patients even when the disease had gone into remission.

It has been reported that positive ASST also correlated strongly with patients who have multiple intolerances to nonsteroidal anti-inflammatory drugs[24] and in some cases may be related to the presence of *Helicobacter pylori* (*H. pylori*) IgG antibodies. The relationship between *H. pylori* and CIU has been reviewed.[25]

### Confirmatory in vitro tests for CAU

Because immunoreactive but non-histamine releasing anti-FcεRI autoantibodies are also detectable in sera from some patients with autoimmune connective tissue diseases, and in a small proportion in patients with other types of urticaria, immunoassays for CAU antibodies have poor specificity. At present the most useful *in vitro* method for the diagnosis of CAU antibodies is the demonstration of the release of histamine (or another reactant) from target basophils or dermal mast cells.[26] At this point in time, the gold standard for detecting clinically relevant autoantibodies to FcεRI is the functional *in vitro* donor basophil histamine release assay. Several laboratories now offer the basophil histamine release assay as a commercial service.

### Other laboratory findings in CAU

Patients with CAU had a significantly lower serum IgE than those with no autoantibody. The significance of this finding is unclear.

That basopenia is a feature of CAU has long been recognized.[4] Histological studies of wheals suggest that one reason for these low values may be the sequestration of basophils within the affected skin.[28] Enumeration of blood basophils, possibly by an automated method might form the basis of a screening test for CAU.

In recent times, an alternative screening test has been proposed based upon the observation that sera from ASST-positive patients with CAU when incubated with basophils from an atopic donor cause CD63 expression on the target basophils.[29] Similarly, donor basophil CD203c expression appears to correlate with autologous serum skin test positivity.[30] Expression of these basophil activation markers can be detected using flow cytometry. This may be developed as a screening test for CAU antibodies, but as yet still requires validation against the basophil histamine release assay.

### Histology

The histological appearance of the skin in CIU resembles that of a late phase reaction.[10] Although the infiltrate in patients with CAU is characterized by more prominent granulocyte infiltrate than that of non-autoimmune patients, the frequency of other infiltrating cells is similar in both groups, although there is a slight increase in serum tryptase and cytokines in CAU patients. These small differences are too insignificant to be used as a diagnostic tool.

It must be stressed that most of the above reports are derived from studies of Caucasian patients in occidental countries. The correlations between certain autoantibodies and corresponding diseases while holding good in Caucasian patients, may not be applicable to Asians.

## CAU and Thyroid Disease

The frequency with which autoimmune thyroid disease is associated with CU has already been remarked upon.[5] The strong association between autoimmune thyroid disease and CAU is also an area of interest in CAU research. There is a generally recognized association between CIU and autoimmune thyroid disease.[31,32] In a recent report, half of the 182 Caucasian (UK) patients with CIU had a positive ASST and there was clear evidence of an association of CAU with thyroid microsomal autoantibodies. These autoantibodies were present in 36/182 CIU patients with cosegregation of anti-FcεRI autoantibodies. Most of these patients are euthyroid, but occasionally, they are hyper- or hypothyroid.[33] Although claims have been made that correcting thyroid dysfunction, if present, also ameliorated the associated urticaria, the evidence is not convincing. However, the association between thyroid disease and CU is sufficiently strong, to prompt adding measurement of the plasma level of thyroid stimulating hormone level (as a screen for thyroid dysfunction) and thyroid autoantibodies to the routine workup of CU patients.[34]

## CAU in Singapore

The prevalence of CAU in Singapore has been found to be about 42%. This does not differ from the established findings. However, thyroid autoimmunity in our patients with CIU appears to be higher than that reported in Western countries. Twenty-one percent of our CIU patients with positive ASST and 24% with negative ASST had serological evidence of thyroid autoimmunity. This number is more than twice that reported in Caucasians. Thyroid autoantibody titers were generally found to be lower in patients with negative ASST compared to those who were ASST positive.

## General Management of CAU

Patients with CU often undergo numerous unnecessary laboratory investigations. A recent study failed to show that increasing the number of laboratory tests would improve the identification of the cause of CU.[35] Guidelines for classification and diagnosis of CU have recently been formulated.[36]

Generally, if no obvious underlying cause for the CU can be found, the patient should be treated with H1 antihistamines supplemented by short tapering courses of prednisolone for flare-ups if necessary. If this is unsuccessful, and the patient's quality of life is impaired, a search for the evidence of CAU should be carried out. Commercial laboratories offering a satisfactory serodiagnostic service for CAU based upon the basophil histamine release assay are available.

Treatment for all forms of CU should include a sound, basic lifestyle. These include avoidance of stress, overtiredness, alcohol, NSAIDS, and tight fitting garments. Nocturnal pruritus can be reduced by tepid showering and keeping the ambient temperature of the bedroom cool. Cooling lotions such as calamine with 1% menthol are popular with patients. H1 antihistamines remain the cornerstone of drug treatment for all types of CU.

## Treatment of CAU: Special Considerations

Patients with proven CAU are initially treated in exactly the same way as patients with CIU. H1 antihistamines remain the initial treatment of choice. The newer second-generation compounds appear to be safe and effective even in off-label dosages.

The treatment of CIU has recently been reviewed.[37,38] After making the diagnosis of CIU, patients with CAU should initially be treated in the same way as those without autoantibodies.

### Oral antihistamines

This includes the use of low-sedation H1 antihistamines in licensed dosages. It is recommended that the period in which the patient is maximally symptomatic be identified, in order to fine-tune the antihistamine therapy to the patient's individual requirements. Most patients experience maximum pruritus at night, so a sedating antihistamine such as hydroxyzine 10-25 mg at night is recommended. However, the patient should be warned about impairment of cognitive function the following day even though there may be no overt symptom of sedation. Dosage of low-sedation antihistamines above those licensed could be tried in non-responders, for example, fexofenadine 360mg daily. Doxepin 10-30 mg can be used instead of night time hydroxyzine.

### Leukotriene antagonists

Early enthusiasm about the value of montelukast, a leukotriene antagonist, has not been borne out by subsequent studies, although it may be of benefit in a small number of patients.

It is not uncommon to find patients to be well controlled most of the time by these measures, punctuated by occasional distressing flares, due very often to personal, occupational, or economic factors, or other previously described exacerbating factors. These relapses are best controlled by brief courses of oral steroids. Prednisolone 30mg daily for five days followed by tapering over five to six days is usually effective, after which the patient can continue regular antihistamines.

### Cyclosporine

Unfortunately, patients with CAU often have severe disease and are poorly responsive to antihistamines, even in high off-label dosages. Cyclosporine is effective and can be used in selected cases of CAU. Cyclosporine has been seen to be effective in selected patients who were ASST positive, in a double-blind placebo-controlled study.[39] Cyclosporine has also been seen to inhibit histamine release from basophils, triggered *in vitro* by sera from patients with CIU known to have anti-FcεRI autoantibodies. The recommended dose range is 4-6 mg/kg/day in addition to oral antihistamines. It is recommended that patients be treated up to three months. About 80% of the patients experience total or partial remission. In these, withdrawal of cyclosporine is followed by sustained remission in about one third. One third of the patients have a relapse, but they are responsive to regular dosages of H1 antihistamines. Although the remaining one third relapse to a severity approximating that suffered before initiating cyclosporine,[40] these patients can be offered further cyclosporine treatment. All patients receiving cyclosporine must be regularly monitored for renal function, serum cholesterol, triglycerides, and blood pressure.

Other immunomodulators that have been reported effective in selected patients with CAU include intravenous immunoglobulin and plasmapheresis.[41]

Other immunosuppressive measures that can be considered, and which have been shown to be helpful in small studies or case reports, include, tacrolimus, methotrexate, and hydroxychloroquine.

A recent report indicated that biologics may be effective in treating recalcitrant CAU. Treatment of allergic asthma with omalizumab produces rapid reduction in free IgE levels and a subsequent decrease in FcεRI expression on mast cells and basophils. If this occurs in CAU, cross-linking of IgE receptors by an autoantibody would be less likely, reducing cell activation and urticaria/angioedema. Twelve patients with CAU, identified by basophil histamine release assay and ASST, with persistent symptoms for at least 6 weeks despite antihistamines, were treated with placebo for 4 weeks followed by omalizumab every 2 or 4 weeks for 16 weeks. The findings suggest that omalizumab is an effective therapy for CAU resistant to antihistamines.[42]

## Conclusion

On the current evidence, the pathogenic role of anti-FcεRI and anti-IgE autoantibodies in CAU is probable, but several problems remain unsolved. CAU fits many, but not all the criteria for autoimmune disease, but the pathogenic factor in patients without autoantibodies remains a mystery. Additionally, the detection of patients with autoantibodies remains a major stumbling block. The ASST is characterized by moderate sensitivity and specificity, but it is an indicator of the presence of circulating vasoactive factors and not specifically autoantibodies. Functional *in vitro* tests suffer from variability between donor basophils or mast cells, and immunoassays detect autoantibodies that may or may not be functional, and so are less specific. There is a need to develop a reliable and easily accessible *in vitro* test.
